# Platelet levels and age are determinants of survival after mild–moderate TBI: A prospective study in Spain

**DOI:** 10.3389/fpubh.2023.1109426

**Published:** 2023-03-20

**Authors:** Oriol Yuguero, Ana Vena, Maria Bernal, Montserrat Martínez-Alonso, Joan Farre, Francisco Purroy

**Affiliations:** ^1^ERLab Emergency Research Group, Institute for Biomedical Research Dr. Pifarré Foundation, IRBLleida, Universitat de Lleida, Lleida, Spain; ^2^Faculty of Medicine, University of Lleida, Lleida, Spain; ^3^Clinical Laboratory, University Hospital Arnau de Vilanova, Lleida, Spain; ^4^Systems Biology and Statistical Methods for Biomedical Research Group, Institute for Biomedical Research Dr. Pifarré Foundation, IRBLleida, Universitat de Lleida, Lleida, Spain; ^5^Neurosciences Group, Institute for Biomedical Research Dr. Pifarré Foundation, IRBLleida, Universitat de Lleida, Lleida, Spain

**Keywords:** TBI, survival, geriatric, emergency, falls

## Abstract

**Introduction:**

Traumatic brain injury (TBI) is a very important reason for consultation in emergency departments.

**Methods:**

A hospital cohort study with patients who attended a hospital emergency department between June 1, 2018 and December 31, 2020 due to TBI was studied. Clinical and sociodemographic variables were recorded. The levels of biomarkers and management variables were used. Qualitative variables were analyzed using Pearson's chi-square test, and quantitative variables using the Mann–Whitney *U*-test. Survival analyses were performed by fitting a multivariable Cox regression model for patient survival during the follow-up of the study in relation to the patient's characteristics upon admission to the emergency department.

**Results:**

A total of 540 patients were included. The mean age was 83 years, and 53.9% of the patients were men. Overall, 112 patients (20.7%) died during the study follow-up. The mortality rate per 100 person-years was 14.33 (11.8–17.24), the most frequent mechanism being falls in the home, with none caused on public roads. The multivariable Cox proportional hazards model showed that survival after TBI was significantly associated with age, S100 levels, Charlson index, patient's institutionalized status, the place where the TBI occurred, and hemoglobin and platelet levels.

**Discussion:**

The most common profile for a patient with a TBI was male and aged between 80 and 90 years. The combination of the variables age, Charlson index, place of TBI occurrence, and hemoglobin and platelet levels could offer early prediction of survival in our population independently of TBI severity. With the data obtained, a therapeutic algorithm could be established for patients suffering from mild TBI, allowing the patient to be supervised at home, avoiding futile referrals to emergency services.

## Introduction

Traumatic brain injury (TBI) is a very important reason for consultation in emergency departments and has increased significantly in recent years, especially in patients over 65 years of age.

A study in 2019 (1) described a change in the characteristics of patients suffering from this type of trauma in Spain. The affected population was older, even for the most severe TBIs, with falls being the most frequent etiology, followed by traffic accidents. Aging is associated with a lower number of interventions and a greater number of sequelae in the days following TBI.

In Spain, the annual incidence of TBI is estimated at 200 new cases per 100,000 inhabitants (2). Of these, 70% recover favorably, 9% die before reaching the hospital, and 6% do so during hospital admission. It is estimated that 15% of cases present a severe functional disability.

A recent European (3) study showed that TBI continues to be a very frequent reason for ED visits and that mortality remains below 10%. Moreover, the study showed that just over 60% of the patients with moderate/severe TBI, and 22% of patients with mild TBI received inpatient rehabilitation, especially those patients with little family support and social and health resources.

Mortality is mainly associated with the characteristics of the TBI and its severity and the patient's baseline situation, or the parameters of systemic severity, as described in a previous study (4). Evolution and recovery are worse if there is a history of cognitive impairment (4), mental disorders, or alcohol use disorders (5, 6). In addition, recent studies show that TBI may be a predisposing factor for developing cardiovascular disease (7).

The use of biomarkers in patients suffering from TBI is increasingly widespread (8), and we believe that in the near future, new TBI management guidelines will include the use of brain damage biomarkers such as S100 in their algorithms (9).

In our setting, faced with a majority of mild TBIs in elderly patients, we approached this study with the aim of finding out what factors could determine mortality in these patients. We wish to examine whether new biomarkers can be predictors of mortality in TBI and whether patient comorbidities influence these biomarker levels and mortality. Our main hypothesis is that biomarkers will change the clinical handling and management of patients with TBIs, especially older patients, and this will lead to a very important change in the observation and follow-up times of these patients upon discharge.

## Materials and methods

This hospital cohort study included patients who attended the hospital's emergency department between June 1, 2018 and December 31, 2020.

### Setting

The Arnau de Vilanova University Hospital is the reference Hospital of a health region with ~400,000 people as it is the only public hospital in the region that treats general emergencies. Patients can attend the emergency department either on their own initiative or after being referred by one of the region's 23 primary care centers. There are three hospitals in the Pyrenees region. Patients requiring further interventions may be referred to a third-level hospital in Barcelona.

### Sample size

According to several studies, 15% of the population included in the study was expected to experience long-term complications. A sample size of 504 patients (63 in group 1 and 441 in group 2) achieved 80% power to detect a difference between the group proportions of 0.1500. The proportion in group 1 (the treatment group) was assumed to be 0.1500 under the null hypothesis and 0.3000 under the alternative hypothesis.

The proportion in group 2 (the control group) was 0.1500. The test statistic used was the two-sided *Z*-test with pooled variance as the standard method in sample size calculation when comparing two independent proportions. The significance level of the test was targeted at 0.0500. The tool used for sample size calculations was PASS software, version 13 (10).

### Inclusion criteria

The main criterion for inclusion was the severity of the TBI, which was determined upon the arrival of the patient by the outcome of the Glasgow Coma Scale (GCS) (11). A patient is deemed to have a severe or grade 2 TBI when scoring ≤8 points on the GCS. Grade 1 TBI is a moderate TBI, in which the patient scores between 9 and 13 points on the scale and presents an increased risk of destabilization. Patients with mild or grade 0 TBI have a GCS score of 13 or higher upon arrival, without any risk factors.

In our study, we included all patients with mild, moderate, and severe TBI who agreed to participate in the study.

### Exclusion criteria

Patients with a cranial or a facial contusion and those who did not give their consent for inclusion were excluded. In addition, subjects who were unable to sign the informed consent or did not understand its purpose were not eligible to participate. Finally, patients who had suffered a TBI in the last 6 months were excluded.

### Recruitment

**Figure 1:**
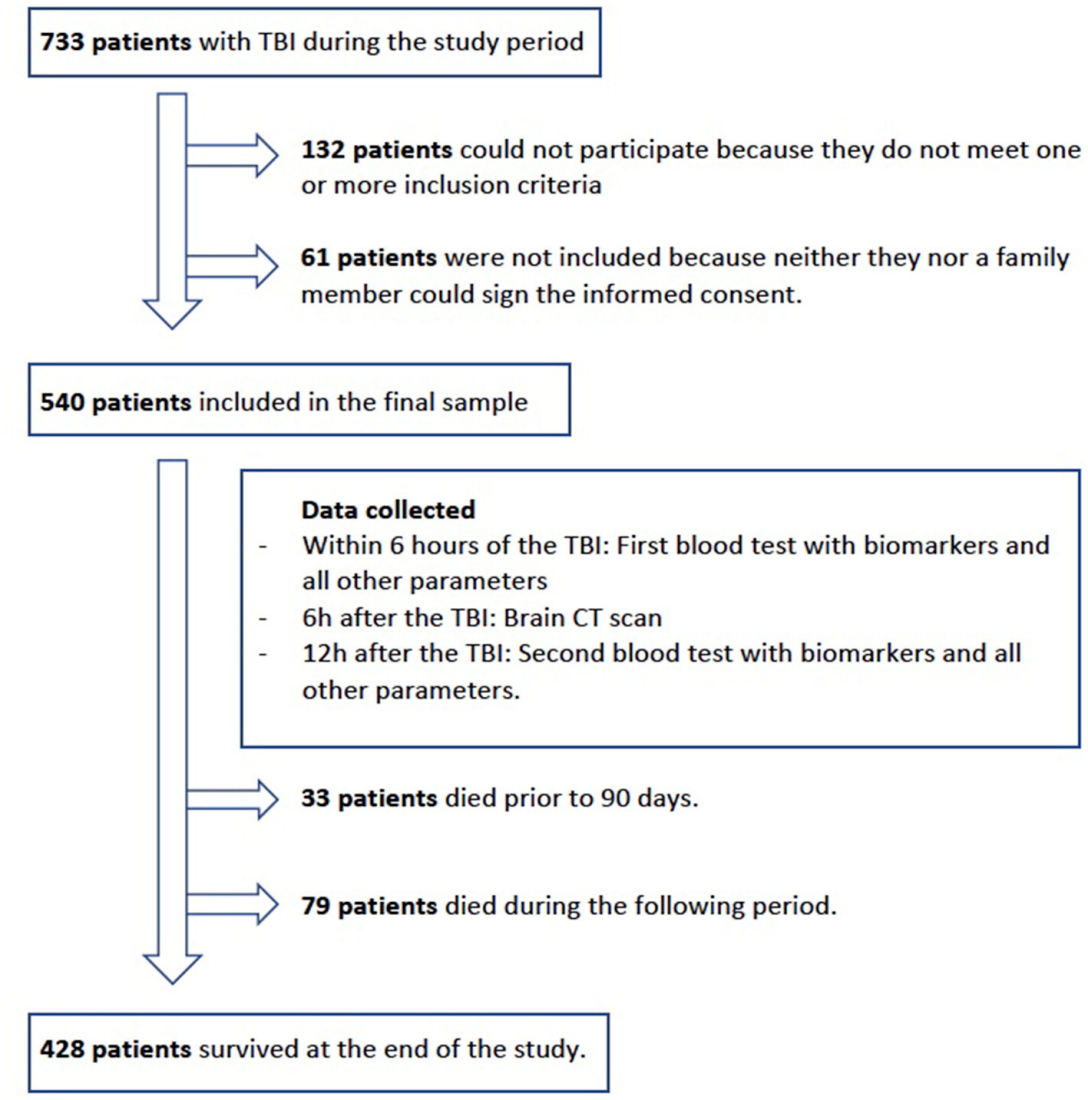
Flowchart of the patients included in the study.

All patients who, upon arrival at the emergency department, reported having suffered a head injury were invited to participate in the study. After assessment by the nursing team, patients not meeting one or more of the criteria or who could not give their consent were not included in the study. Figure 1 shows patient inclusion flow.

### Variables

To carry out this study, different variables were collected to assess the patients' baseline situations and whether there were any significant analytical alterations associated with the prognosis of TBI.

- Clinical: To evaluate the impact of the patients' clinical situations, we selected their baseline situation by means of the short-form Charlson index, which has been used in other studies (12) on patients with trauma. In addition, we took into account the cardiovascular history of the patients, such as antihypertensive, antidiabetic, or lipid-lowering treatments, in view of this new profile of patients suffering from a TBI who could end up developing cardiovascular disease.
- Complementary tests were performed on any patient with trauma in our hospital which included blood tests (with hemoglobin levels, platelets, and INR) upon patient arrival and 12 h after the TBI episode, as well as a cranial CT with its findings.
- Biomarkers linked to brain damage: The marker S100B has been used in multiple studies due to its relationship with the prognosis of TBI (13), and other markers such as UCHL1, NSE, and GFAP levels have been associated with the onset of complications in TBI (14). We obtained the values of these markers at 6 h and 12 h after the TBI, as they are the minimum observation times at our center.
- Sociodemographic variables: Age, gender, place of residence, and place where the TBI occurred, as well as whether polytrauma had been associated with TBI.
- Management variables: Time of arrival, time of discharge, observation time, and discharge destination to ascertain the management of these patients and, in particular, to find out how many would require social resources upon discharge.
- Survival after the TBI. Follow-up of patients up to 18 months after TBI. For the survival analysis, we recorded death of the patient during that period as the failure event.

### Statistical analysis

For quantitative variable description, the median and the 25th and 75th percentiles were calculated, while for qualitative ones, absolute and relative percentages were used. Qualitative variables were compared between survivors and non-survivors using Pearson's chi-square test, while quantitative variables were compared with the Mann–Whitney *U*-test.

A multivariable Cox proportional hazards model for patient survival during the follow-up of the study was adjusted in relation to a subset of sociodemographic, clinical, and analytical variables. This subset was selected based on the results of the Boruta algorithm (15) for the selection of important features beyond the estimated importance achievable at random using permutated copies of the original data. Only the variables with a significant coefficient were kept in the model. The possible interaction of these variables with sex was tested and taken into account in the final model if statistically significant. Statistically significant quantitative explanatory variables were standardized to interpret their associated hazard ratios per one standard deviation (SD) regardless of their different units. The hazard ratios provided were estimated from the model, with a significant interaction between sex and S100. This model included a coefficient for male sex (reference: female), a coefficient for S100, and a coefficient for the interaction between both variables. Thus, exp (S100 coefficient) was the estimated HR associated with one SD of S100 (previously standardized) if the sex was female, while exp (S100 coefficient + interaction coefficient) was the estimated HR associated with one SD of S100 if the sex was male. In this way, the HR associated with S100 was estimated depending on the sex of the patient. The proportional hazard assumption was verified for the final Cox model (16). The proportional hazards hypothesis can be assumed for the model as a whole and for all the coefficients of the model. R software was used (17), and a significance level of 5% was applied.

### Ethical aspects and information management

All clinical information was obtained during the emergency department visit for the TBI episode. A patient follow-up to determine patient survival was obtained using SAP software. The information was collected by the research team. Patient results and information were managed according to the recommendations of our ethics committee.

The study was approved by the CEIC of the Hospital Universitario Arnau de Vilanova de Lleida (CEIC-1952). All patients included in the study, or their legal representatives, consented to participate in the study prior to initiating the study in the emergency department. The processing, communication, and transfer of the personal data of all participating subjects complied with the provisions of Spanish Organic Law 3/2018, on the Protection of Personal Data and Guarantee of Digital Rights (LOPD-GDD 3/2018) and Regulation 2016/679 (EU) of the European Parliament and of the Council of Europe of 27 April 2016.

## Results

The total sample of patients was 540. The description of the sample can be seen in Table 1. The diagram in Figure 1 shows the patients who were excluded from the study. A total of 193 patients with TBIs were excluded, of which 132 patients did not meet one or more of the inclusion criteria (mainly contusions to the facial region or parietal without being a true case of TBI), and 61 patients were not included because neither they nor a family member could sign to give informed consent. The mean age was 83 years, with 53.9% of the patients being men. Approximately 71.7% of TBIs occurred in the patient's home. A total of 18.5% of the patients had been diagnosed with dementia.

**Table 1:** Sample characteristics.

	**[All]**	**Survivor** **428 (79.2%)**	**Death** **112 (20.9%)**	***p*. overall**
Age	83.0 [73.0; 88.0]	82.0 [68.8; 87.0]	86.0 [83.8; 90.0]	<0.001
**Gender**				
Female	249 (46.1%)	201 (80.7%)	48 (19.3%)	0.503
Male	291 (53.9%)	227 (78.0%)	64 (22.0%)	
**Place of TBI**				
Place of residence	387 (71.7%)	287 (74.2%)	100 (25.8%)	<0.001
Outside	153 (28.3%)	141 (92.2%)	12 (7.84%)	
**Place of residence**				
Home	480 (88.9%)	391 (81.5%)	89 (18.5%)	0.002
Geriatric center	56 (10.4%)	35 (62.5%)	21 (37.5%)	
Sociosanitary home	4 (0.74%)	2 (50.0%)	2 (50.0%)	
Smoker	20 (3.70%)	15 (75.0%)	5 (25.0%)	0.582
Alcohol consumer	19 (3.52%)	15 (78.9%)	4 (21.1%)	1
Drug abuse	13 (2.41%)	12 (92.3%)	1 (7.69%)	0.321
**Comorbidities**				
Charlson index	2.00 [1.00; 3.00]	2.00 [1.00; 3.00]	3.00 [2.00; 4.00]	<0.001
Dementia	100 (18.5%)	63 (63.0%)	37 (37.0%)	<0.001
Hypertension	423 (78.3%)	321 (75.9%)	102 (24.1%)	<0.001
Diabetes mellitus	171 (31.7%)	131 (76.6%)	40 (23.4%)	0.357
Dislipemia	204 (37.8%)	163 (79.9%)	41 (20.1%)	0.859
**Treatment**				
Antihipertensive	396 (73.3%)	300 (75.8%)	96 (24.2%)	0.001
Antidiabetic	137 (25.4%)	108 (78.8%)	29 (21.2%)	0.983
Insulin	45 (8.33%)	33 (73.3%)	12 (26.7%)	0.405
Statins	163 (30.2%)	127 (77.9%)	36 (22.1%)	0.696
Anticoagulant	216 (40.0%)	169 (78.2%)	47 (21.8%)	0.713
Antiplatelet	190 (35.2%)	142 (74.7%)	48 (25.3%)	0.072
**Clinical/analytical variables**				
Systolic blood pressure	144 [129; 163]	143 [129; 163]	144 [128; 165]	0.979
Diastolic blood pressure	77.0 [67.0; 86.0]	77.0 [69.0; 87.0]	72.0 [64.0; 84.2]	0.002
Heart rate	74.0 [65.0; 86.0]	74.0 [65.0; 88.0]	71.5 [66.0; 81.0]	0.147
Temperature^*^	36.0 [35.5; 36.3]	36.0 [35.5; 36.3]	36.0 [35.6; 36.3]	0.559
No pupillary alteration	535 (99.1%)	425 (79.4%)	110 (20.6%)	0.278
Hb^*^	12.9 [11.7; 14.1]	13.1 [12.0; 14.3]	12.4 [10.6; 13.6]	<0.001
Platelets^*^	204 [160; 246]	203 [162; 244]	209 [152; 264]	0.4
INR	1.13 [1.03; 1.67]	1.13 [1.03; 1.65]	1.15 [1.07; 1.80]	0.167
Cranial CT realization	537 (99.4%)	425 (79.1%)	112 (20.9%)	1
Normal CT	326 (60.4%)	265 (81.3%)	61 (18.7%)	0.185
Acute pathology in CT	74 (13.7%)	55 (74.3%)	19 (25.7%)	0.331
**TBI**				
Mild	505 (93.5%)	399 (79.0%)	106 (21.0%)	0.095
Moderate	20 (3.70%)	19 (95.0%)	1 (5.00%)	
Severe	15 (2.78%)	10 (66.7%)	5 (33.3%)	

* 1 missing value.

Quantitative variables are described using the median and the 25th and 75th percentiles and compared using the Mann–Whitney U-test. Qualitative variables are described using absolute and relative frequencies and compared using Pearson's chi-squared test unless there were expected frequencies lower than 5, in which case the Fisher exact test was used.

**Figure 2:**
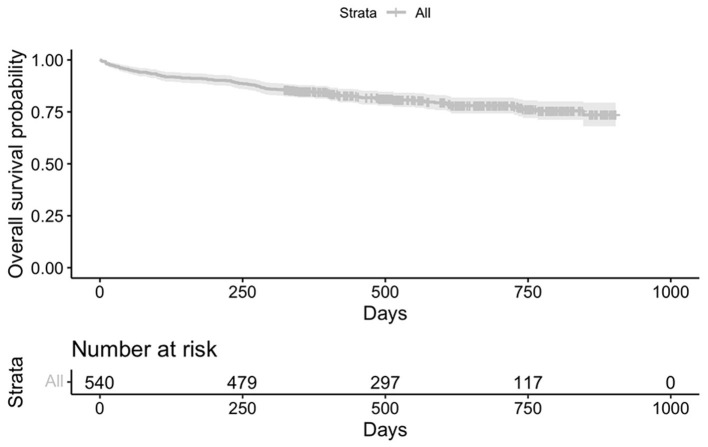
Kaplan–Meier survival curve along the follow-up period.

A total of 112 patients (20.7%) died during the study follow-up. The maximum follow-up recorded was 903 days, with a median of 522 days. Follow-up is understood as the monitoring of patients throughout the study period. We differentiate an initial period covering the first 90 days, and a second period until the end of the study. Figure 2 shows the overall Kaplan–Meier survival curve to illustrate the survival decrease during the follow-up period.

A total of 33 patients died (6.53%) between the time of admission and 90 days later. No deaths were recorded after 90 days for cases of TBIs occurring on public roads or in accidents; hence, deaths occurring in the first days or week were recorded.

Of the sample, 93.5% had a mild or grade 0 TBI, while 2.7% had a severe TBI. Of all the patients, 80.9% returned home after an average observation time of 11.4 h in the emergency department. As for cardiovascular risk factors, 78.3% had hypertension and <5% of patients were smokers. Regarding anticoagulant treatment, 40% of the patients received such treatment, while 35.2% received antiplatelet therapy.

Reported levels of Hb (12–16 gr/dl) and INR (0.8–1.2) were within conventional thresholds (18). Mean platelet levels were 204,000 (39,000–785,000).

**Figure 3:**
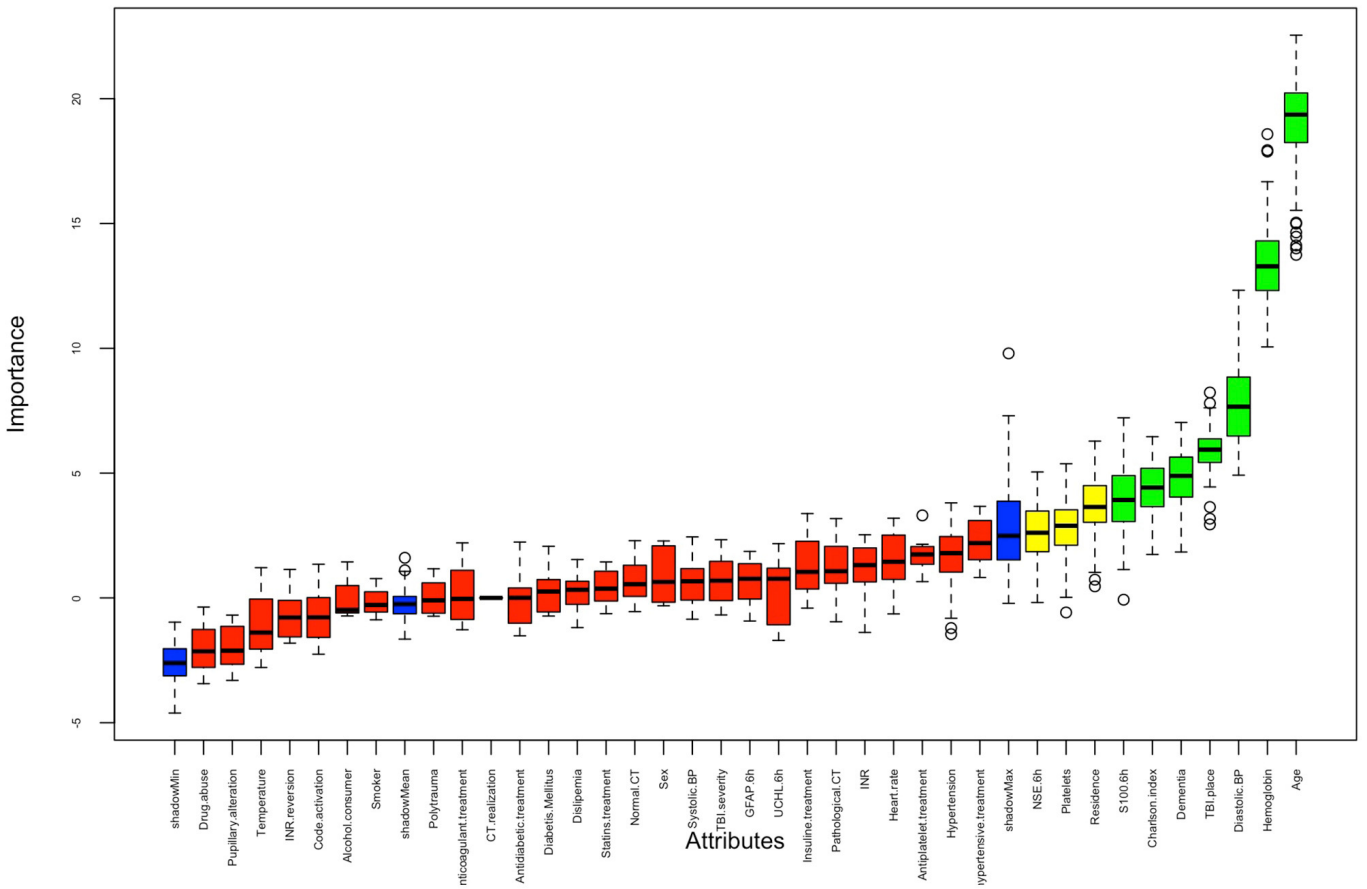
Borutha algorithm to select explanatory variables.

Bivariate analysis showed a statistically significant relationship between survival and age, Charlson index, presence of dementia, place of residence, place of TBI, and levels of hemoglobin and diastolic blood pressure. To select the most important explanatory variables, the Boruta algorithm was used (Figure 3), which selects the same variables, but adds platelet levels and the biomarkers S100 and SES levels. Boruta's algorithm shows the relationship between the different variables with our survival outcomes. The green color shows the variables that reveal a confirmed important relationship with survival, and the red shows the variables that have no relationship. For the ones in yellow (labeled as tentative), there is insufficient evidence to reject or confirm them, while the blue ones represent the minimum, mean, and maximum importance of the “shadow features” generated by this method by randomly shuffling each original variable.

The final multivariable Cox proportional hazards model without biomarkers and after checking for possible interactions with gender shows a significant association of gender (with no significant interaction), age, place of TBI occurrence, Charlson index, diastolic blood pressure, and Hb levels with the survival of patients with TBIs (Table 2). Thus, the model adjusted for all these variables shows that high baseline levels of diastolic blood pressure or Hb are associated with higher survival rates, while a high Charlson index and advanced age are associated with lower survival. In addition, men show a higher likelihood of death than women, and survival varies according to the place where the TBI took place. TBIs in residential/healthcare settings show a worse survival rate than TBIs occurring in the home and on public roads.

**Table 2:** Cox proportional hazards model 1 (without biomarkers) and model 2 (with significant biomarkers, NSE and S100).

**Characteristic**	**Model 1**			**Model 2**		
	**HR** ^a^	**95% CI** ^a^	***p*** **-value**	**HR** ^a^	**95% CI** ^a^	***p*** **-value**
Male (ref. female)	1.95	1.31, 2.89	<0.001	2.10	1.41, 3.12	<0.001
Age^*^	2.29	1.53, 3.42	<0.001	2.54	1.67, 3.86	<0.001
Hemoglobin^*^	0.78	0.64, 0.95	0.012	0.75	0.62, 0.92	0.012
DBP^*^	0.78	0.63, 0.96	0.022	0.79	0.64, 0.98	0.022
**Place of TBI** ^**^ **(ref. at home)**						
In an institution	1.60	0.98, 2.62	0.060	1.60	0.98, 2.61	0.062
Outside their residence	0.49	0.26, 0.91	0.025	0.48	0.26, 0.90	0.022
Charlson index^*^	1.27	1.05, 1.53	0.014	1.24	1.02, 1.50	0.027
NSE^*^				1.16	1.02, 1.33	0.024
**S100** ^*^ **depending on sex**						
S100^*^ if female				0.80	0.52, 1.22	0.296
S100^*^ if male				1.49	1.19, 1.85	<0.001

a HR, hazard ratio; CI, confidence interval.

* Standardized variable.

** Recoded site of TBI (traumatic brain injury) occurrence including place of residence (they are strongly related); ref. reference category; model.

If we add biomarkers to the model, a statistically significant interaction between S100 levels and sex is detected. Thus, higher levels of S100 are associated with significantly higher mortality in men. Regarding the NSE, higher levels are associated with higher mortality.

## Discussion

During the 2-year study period, we observed that in our population, the most common profile for a patient with a TBI was male and within the age range of 80–90 years. This demographic profile corresponds with previous research (19), and it should come as no surprise that this patient profile will probably increase.

The most common mechanism was falls in the home. These are considered to be the most frequent cause of accidental death in the elderly. Approximately 8% of people over 65 years of age visit hospital emergency departments annually due to falls. Once an elderly person falls, the risk of a second episode increases exponentially (20).

Hypertension, the use of anticoagulants, and underlying cognitive disorders were the most frequent pathologies associated with the history of our sample (21). Our data agree with those of Hawley et al. (22) and Gardner et al. (23), in that the most frequent comorbidity was hypertension.

The highest percentage of our patients suffered a mild TBI and were discharged home with a mean stay of 11.4 h in the emergency observation area. Our high percentage of mild TBI is in contrast to what is described in the literature since previous studies have indicated that in most cases, patients with mild TBI cases do not present at emergency departments (24). This may be related to the differences in health coverage between countries. However, it may also be due to the lack of uniform criteria for the definition of mild head injury, and on the contrary, not all patients who suffer this type of accident are reflected in the healthcare statistics (25).

The mortality rate per 100 person-years was 14.33 (11.8–17.24), the most frequent mechanism being falls in the home, with none taking place on public roads. This analysis corroborates the findings of the study by Peterson and Kegler (26), which showed an increase in TBIs due to falls and an increase in the mortality rate for falls compared to other mechanisms.

The age of a patient who suffers from a TBI is the variable to which most attention has been paid in the various studies (27, 28). Regarding its relationship with survival, the initial results of the Glasgow and Rotterdam groups suggested that mortality increased exponentially with the age of the patient at the time of the initial injury. In our sample, we also found a statistically significant relationship between lower survival and increasing age.

Other independent factors associated with mortality were lower diastolic blood pressure, the presence of dementia, pupillary disturbances, or a high Charlson index. It seems logical that a higher Charlson index score should correlate with higher mortality since in more complex patients, TBI can be a destabilizing element that can alter the patient's multimorbidities. It is precisely these types of users who tend to be institutionalized, so it is consistent that patients who reside in nursing homes or residences have lower survival rates.

On the contrary, it was found that the combination of the variables age, a high Charlson index, place of TBI occurrence, and hemoglobin and platelet levels (28) could predict early survival in our population, mainly in patients with mild TBI. In the specific case of mild TBI, a higher Charlson index and age were associated with lower survival, while higher baseline diastolic blood pressure and Hb levels were associated with longer survival.

Various publications have previously pointed out that the presence of prehospital hypotension and hypotension on arrival at the hospital is associated with the development of secondary injury (29) and increased mortality in TBIs in the elderly population. Low hemoglobin levels could jeopardize the oxygen supply to the brain and the tissue response to acute brain injury (30).

In terms of platelet levels, thrombopenia has been reported to increase mortality in TBI (31), especially in moderate–severe TBI. Thrombopenia facilitates bleeding and the progression of cerebral edema, which leads to an increase in intracranial pressure and a decrease in cerebral perfusion pressure (32).

Finally, higher levels of NSE and S100 in men are associated with a worse prognosis and mortality. S100 had already been described as a prognostic biomarker of severity and mortality (33) and recently, also in NSE (34). However, we have not found any association with the rest of the biomarkers.

The main limitation of our study is that in our sample, a high percentage of patients had a mild TBI, because they were the group of patients with TBIs who attended our emergency department, and this could lead to a selection bias. However, this may be a strength as we have a highly representative sample of the group of elderly patients with TBIs that we usually treat in the hospital's emergency department. We believe that the patients' ages could also lead to a bias, as it would be interesting to know whether the results obtained would be similar in a sample of younger patients.

In conclusion, we know that the age and multimorbidity of the patient, which may condition their frailty, are fundamental factors in survival after TBI and that hypotension and thrombopenia may be associated with increased mortality. We also know that serum biomarkers can be useful, but other variables must be taken into account.

We believe that with the data obtained, our study may have clinical and management implications. Regarding clinical applications, we believe that a therapeutic algorithm could be established for patients suffering from mild TBIs, especially those who are in residences (this could be considered discriminative), in which, depending on the variables obtained, the patient could be supervised at home, avoiding futile referrals to emergency services. Moreover, we hope that our results might prove useful to community health professionals who act in the direct care of the elderly who suffer falls, as well as their families and residential care workers, to highlight the need to establish a community health policy for the prevention of frailty (functional, cognitive, and social) or, as Thompson et al. (35) described it, to prevent one of the “epidemics of frailty” and one of the “silent epidemics” of our century: elderly patient falls. Finally, we believe that these new protocols and the search for tools that facilitate at-home patient supervision might improve their comfort and convenience, and this will lead to an improvement in the quality of care for patients suffering from TBIs.

## Data availability statement

The raw data supporting the conclusions of this article will be made available by the authors, without undue reservation.

## Ethics statement

The studies involving human participants were reviewed and approved by CEIm Hospital Universitary Arnau de Vilanova de Lleida. The patients/participants provided their written informed consent to participate in this study.

## Author contributions

OY and FP lead the research. MB and JF did the lab tests and analysis. AV did the data base. MM-A performed the statistical analysis. All authors contributed in the paper draft and reviewed the final version. All authors contributed to the article and approved the submitted version.
